# Development of Gluten-Free Corn Snacks Enriched with White Mulberry Fruit: Polyphenolic Composition, Antioxidant Activity and In Vitro Gastrointestinal Stability of Phenolic Compounds

**DOI:** 10.3390/molecules31132370

**Published:** 2026-07-05

**Authors:** Kamila Kasprzak-Drozd, Agnieszka Ziółkiewicz, Karolina Wojtunik-Kulesza, Marek Gancarz, Iwona Kowalska, Justyna Misiurek, Magdalena Wójciak, Ireneusz Sowa, Tomasz Oniszczuk, Maciej Combrzyński, Anna Oniszczuk

**Affiliations:** 1 Department of Inorganic Chemistry, Medical University of Lublin, Chodźki 4a, 20-093 Lublin, Poland; kamila.kasprzak-drozd@umlub.edu.pl (K.K.-D.); ziolp@o2.pl (A.Z.); karolina.wojtunik-kulesza@umlub.edu.pl (K.W.-K.); justyna.misiurek@umlub.edu.pl (J.M.); 2 Faculty of Production and Power Engineering, University of Agriculture in Krakow, Balicka 116b, 30-149 Krakow, Poland; m.gancarz@ipan.lublin.pl; 3 Institute of Agrophysics Polish Academy of Sciences, Doświadczalna 4, 20-290 Lublin, Poland; 4 Department of Phytochemistry, Institute of Soil Science and Plant Cultivation, State Research Institute, 24-100 Puławy, Poland; ikowalska@iung.pulawy.pl; 5 Department of Analytical Chemistry, Medical University of Lublin, Chodźki 4a, 20-093 Lublin, Poland; magdalena.wojciak@umlub.edu.pl (M.W.); ireneusz.sowa@umlub.edu.pl (I.S.); 6 Department of Food Process Engineering, University of Life Sciences in Lublin, Głęboka Street 31, 20-612 Lublin, Poland; tomasz.oniszczuk@up.edu.pl (T.O.); maciej.combrzynski@up.edu.pl (M.C.)

**Keywords:** functional food, extrusion cooking, in vitro digestion, biological activity, antioxidants, polyphenols, *Morus alba* L.

## Abstract

The aim of this study was to evaluate the effect of adding white mulberry (*Morus alba* L.) fruit to extruded corn snacks on their polyphenol profile, antioxidant properties, acetylcholinesterase (AChE) inhibitory activity and the preservation of phenolic compounds in an in vitro digestion model. Mixtures of corn grits with 0, 10, 15 and 20% dried mulberry fruit were extruded at temperatures of 100, 120 and 140 °C, and then the total polyphenol content (TPC) and antioxidant activity (IC_50_ for DPPH) were determined. For selected samples (0%, 140—3E; 15% mulberry, 140—9E; mulberry—13E), further antioxidant tests (FRAP, CUPRAC, Fe^2+^ chelation) were performed, the phenolic compound profile (UHPLC) and AChE inhibition were assessed, and a two-step in vitro digestion was conducted. The addition of mulberry significantly increased TPC- and free-radical-scavenging capacity compared to the control sample, with snacks containing 15% mulberry extruded at 140 °C showing approximately a 3.5-fold higher TPC than the control, while dried mulberry fruit itself exhibited about a five-fold higher TPC than this enriched snack. Among the snacks, the most favorable DPPH-radical-scavenging effect was obtained for the variant with 20% mulberry at 120 °C (IC_50_ = 0.176 mg/mL), whereas the mulberry fruit extract reached an IC_50_ of 0.0926 mg/mL. In a two-step in vitro digestion model, the mulberry-enriched snack with 15% fruit retained 69.3% of its initial TPC after the gastric phase and 33.3% after the intestinal phase, compared with 55.0% and 20.0%, respectively, for the control snack, confirming a partial but meaningful preservation of phenolic compounds under simulated gastrointestinal conditions. UHPLC analysis confirmed that mulberry and the enriched snacks are a rich source of chlorogenic acids and their isomers, as well as quercetin and kaempferol glycosides, which largely survived the two-step in vitro digestion, despite an observed decrease in TPC after the gastric stage and a further reduction after the intestinal stage. At the same time, mulberry extract and mulberry-enriched snacks exhibited high antioxidant activity in all tests conducted and in vitro AChE inhibitory activity, suggesting that *Morus alba* L. fruit has the potential to be used as a natural functional ingredient in the production of gluten-free snacks with antioxidant and potentially neuroprotective properties.

## 1. Introduction

Oxidative stress is a condition in which there is an excess of free radicals in the body relative to the body’s ability to neutralize them through antioxidants and is associated with the development of many lifestyle-related diseases, such as diabetes, cancer, cardiovascular diseases and neurodegenerative disorders [1]. For this reason, dietary patterns that regularly include foods naturally rich in antioxidants, such as fruits, vegetables, nuts and whole grains, are considered beneficial for maintaining overall health status and supporting normal physiological functions, including those related to the cardiovascular and nervous systems. It should be emphasized, however, that these effects refer to the long-term consumption of a balanced diet rather than to any single food product. In this context, developing snacks with an increased content of bioactive compounds, including polyphenols, may contribute to improving the nutritional profile of gluten-free diets, but the present study focuses on the characterization of their polyphenol composition, antioxidant properties and in vitro gastrointestinal stability rather than on demonstrating direct clinical health outcomes [2].

In recent years, increasing attention has also been paid to the development of gluten-free products with enhanced health-promoting properties. This is particularly important not only for individuals with celiac disease or gluten intolerance, but also for consumers seeking functional foods that combine nutritional value with additional physiological benefits. Gluten-free snacks based on maize are widely consumed; however, they are often characterized by relatively low levels of bioactive compounds. Therefore, their enrichment with plant-derived ingredients rich in antioxidants represents a promising approach to improving their functional quality [3,4].

Polyphenols act as antioxidants in food, exerting their effects through various mechanisms. It is known that plants rich in polyphenols exhibit a diverse redox status [4]. As a result, they can be used as health-promoting additives to enrich food. White mulberry (*Morus alba* L.) is a promising functional ingredient because its fruits contain high amounts of phenolic acids, flavonoids, vitamins and other compounds with antioxidant, anti-inflammatory, hypoglycemic and potentially neuroprotective effects [5,6]. Importantly, some of these compounds have been reported to inhibit acetylcholinesterase (AChE), an enzyme responsible for the breakdown of acetylcholine. AChE inhibition is a key therapeutic target in the management of neurodegenerative diseases, particularly Alzheimer’s disease, as it may enhance cholinergic transmission and support cognitive function. Therefore, the inclusion of AChE-inhibiting compounds in functional foods may contribute to neuroprotective dietary strategies. White mulberry fruits are traditionally used in the prevention of metabolic disorders, particularly diabetes, which provides an additional argument for including them in snack formulations [7,8]. This fits into the broader concept of functional foods, in which food is expected not only to provide nutrition but also to support health maintenance and disease prevention [9].

Extrusion cooking is particularly useful in this type of research because it allows plant-based additives to be incorporated into cereal matrices, while also making it possible to determine whether high temperatures and mechanical shearing preserve or release bioactive compounds [10,11].

The aim of this study was to evaluate the effect of adding white mulberry fruit to extruded corn snacks on their polyphenol profile, antioxidant properties, acetylcholinesterase inhibitory activity and the preservation of phenolic compounds in an in vitro digestion model. Mixtures of corn grits with 0, 10, 15 and 20% dried mulberry fruit were extruded at temperatures of 100, 120 and 140 °C, and then the total polyphenol content (TPC) and antioxidant activity (IC_50_ for DPPH) were determined. For selected samples (0%, 140—3E; 15% mulberry, 140—9E; mulberry—13E), further antioxidant tests (FRAP, CUPRAC, Fe^2+^ chelation) were performed, the phenolic compound profile (UHPLC) and AChE inhibition were assessed, and a two-step in vitro digestion was conducted.

## 2. Results

### 2.1. Effect of White Mulberry Addition and Extrusion Temperature on the Polyphenol Content and Antioxidant Properties of Corn Snacks

ASE was performed on the prepared corn snack samples, followed by subsequent analyses of the obtained extracts [12]. In the first phase of the experiment, the authors examined the total polyphenol content in the analyzed extracts. The results showed that this content increased significantly with the addition of mulberry fruit (Table 1). The highest total polyphenol content (calculated as gallic acid equivalents—GAE) was recorded in snacks containing 20% mulberry fruit, produced at 140 °C, while the lowest was found in the sample without this functional additive.

The next stage focused on the effect of the functional additive and the extrusion temperature on the antioxidant properties of the snacks. The aim of the study was to determine the IC_50_, i.e., the concentration (mg/mL) that causes a 50% decrease in the initial concentration of the DPPH radical. The results (Table 1) showed that all snacks enriched with white mulberry fruit exhibited a greater ability to scavenge DPPH radicals than snacks without fruit. The most favorable IC_50_ was recorded for snacks containing 20% mulberry fruit, produced at 120 °C, while the lowest was observed in the sample without mulberry.

The experiment demonstrated that snacks containing mulberries are an important source of antioxidants. These products are particularly recommended for people on a gluten-free diet, as they do not contain gluten [13]. Snacks with a 15% mulberry fruit content extruded at 140 °C (sample 9E) were selected as the main variant for further detailed analyses. Although the 20% formulation exhibited the highest or among the highest TPC- and DPPH-radical-scavenging activity, the 15% level was considered a practical compromise between a substantial increase in polyphenol content and acceptable technological properties under the applied processing conditions. In preliminary technological observations, snacks with 20% mulberry addition showed a markedly darker, more brownish appearance compared with the 15% variant. A higher load of simple sugars and polyphenols at 20% mulberry could, in principle, promote Maillard-type reactions and oxidative changes; however, no formal shelf-life or lipid oxidation studies were conducted in this work, so these aspects should be regarded as technological considerations rather than experimentally confirmed effects. Therefore, the 15% mulberry formulation was chosen as a representative enriched product for further UHPLC analysis, in vitro digestion and AChE inhibition assays, while recognizing that the 20% variant may provide even higher antioxidant capacity but with less favorable appearance characteristics. As reference samples, we selected snacks without mulberry addition that were produced at the same temperature as the crispies containing 15% mulberry (140 °C, sample 3E) and white mulberry fruit (sample 13E).

The results presented above are significant from a practical standpoint. They indicate that both white mulberries and food products enriched with them are a source of biologically active polyphenols that are partly retained during a high-temperature, short-time extrusion process involving intensive mechanical shear.

### 2.2. UHPLC-DAD-MS Analysis of Polyphenolic Constituents in White Mulberry-Enriched Corn Snacks

The analysis of phenolic compounds was performed based on mass data and UV–Vis spectra of the main peaks recorded in the chromatograms. The compounds listed in Table 2 and Table 3 were identified in the sample without Morus additives (3E), the sample with Morus additive (9E), and the *Morus alba* L. extract (13E).

Chromatographic analysis showed that mulberry fruit is a rich source of various polyphenols, mainly chlorogenic acids and quercetin and kaempferol glycosides. The addition of 15% mulberry to the extrudate allowed for the preservation of the qualitative profile of these compounds, although their concentrations are expected to decrease due to dilution in the cereal matrix and partial degradation during processing. The snack without mulberry addition contained only low levels of a limited set of phenolic acids, indicating that the marked improvement in the polyphenol profile and antioxidant properties of the enriched snacks is largely attributable to the mulberry fruit addition rather than to the corn base alone. Thus, the UHPLC data provide a qualitative–quantitative basis for interpreting the TPC and antioxidant test results and support the selection of the snack variant with a 15% mulberry addition as a compromise between enhanced polyphenol content and acceptable technological characteristics.

In both mulberry-containing samples (9E and 13E), a similar set of major polyphenolic compounds was identified: derivatives of hydroxybenzoic acids (dihydroxybenzoic acid hexoside, protocatechuic acid), numerous chlorogenic acid derivatives (neochlorogenic, chlorogenic, cryptochlorogenic, p-coumaroylquinic, 3,5-dicaffeoylquinic, 4,5-dicaffeoylquinic) and a broad spectrum of flavonol glycosides—mainly quercetin derivatives (quercetin dihexoside, quercetin rhamnosyl dihexoside, quercetin 3-O-glucoside, quercetin acetylglucoside) and kaempferol glycosides (kaempferol 3-O-rutinoside, kaempferol 3-O-glucoside, kaempferol derivative). This spectrum is typical of white mulberries. According to data presented by other authors, the fruit is dominated by chlorogenic acids, rutin and other quercetin glycosides, supplemented by a smaller proportion of kaempferol derivatives [14,15].

### 2.3. Assessment of Antioxidant Properties

#### 2.3.1. Determination of Iron Ion-Reducing Capacity (FRAP)

The determination of the antioxidant properties of food using the FRAP (Ferric Reducing Antioxidant Power) revealed that the sample with a 15% mulberry fruit addition exhibited the highest reducing potential, while the snack without additives and the mulberry fruit extract showed significantly lower values (Table 4).

The determination of the antioxidant properties of food using the FRAP assay revealed that the sample with a 15% mulberry fruit addition exhibited the highest reducing potential, while the snack without additives and the mulberry fruit extract showed significantly lower values (Table 4). Interestingly, under our experimental conditions, the FRAP value for the mulberry extract alone was lower than for the enriched snack, which may be related to matrix effects, rather than to a genuinely lower intrinsic activity of the raw fruit. The starch–protein matrix of the snack may also facilitate the extraction or stabilization of certain reducing compounds that are particularly well detected by the FRAP assay. All tested samples exhibited relevant reducing properties, but their activity remained lower than that of gallic acid used as a positive control, which is consistent with the high reactivity of this low-molecular-weight phenolic standard.

#### 2.3.2. Results of the Determination of Cu^2+^-Reducing Capacity (CUPRAC)

The CUPRAC method (CUPric Reducing Antioxidant Capacity) is one of the most widely used techniques for determining the antioxidant properties of food [15]. For mulberry samples (Table 4), the sample with a 15% mulberry fruit addition exhibited the highest antioxidant potential, while the snack without additives and the mulberry fruit extract showed significantly lower values.

The results of the CUPRAC method indicate that the addition of mulberry significantly increases the ability to reduce Cu^2+^ ions to Cu^+^ compared to the snack without additives, although all samples remain less active than GA used as positive control (data not presented). At the same time, the differences between the mulberry-enriched snack and the mulberry extract alone are relatively small, suggesting that both the raw material and the enriched product retain a similar level of antioxidant “potency” in this method.

The snack with mulberry added exhibits a higher Trolox equivalent than the one without additives, reflecting an increase in the content of polyphenols derived from the fruit. This is consistent with the results obtained in TPC and UHPLC, where it was observed that enriching products with mulberry causes a significant increase in the content of phenolic compounds, especially chlorogenic acids and flavonol glycosides. GA, as a strong, low-molecular-weight antioxidant, achieves values several times higher than the tested extracts.

#### 2.3.3. Results of the Determination of the Chelating Capacity for Fe^2+^ Ions

Fe^2+^ ion chelation is versatile method and can be applied to various types of food. The results clearly show that the mulberry fruit extract exhibits the strongest iron-binding capacity, the sample with a 15% mulberry addition exhibits a slightly weaker capacity, while the snack without additives exhibits by far the weakest capacity (Table 5).

This confirms the key role of mulberry as a source of compounds capable of sequestering transition metal ions, which is one of the important mechanisms of protection against reactive oxygen species. It is worth to underline that extract from mulberry revealed 100% chelation of Fe (II) and this effect was almost entirely retained in food containing a 15% addition of this fruit, as the chelation rate was almost 97%. In practice, this means that mulberries have a very high capacity to bind Fe^2+^ ions, which consequently has a positive effect on the proposed snacks. It should also be noted that there is a significant increase in chelating activity compared with samples without added fruit.

#### 2.3.4. Determination of DPPH-Free-Radical-Scavenging Capacity

The results of the DPPH-free-radical-scavenging capacity assay clearly show that the addition of mulberry increases the antioxidant potential of the snacks, with the dried mulberry fruit extract exhibiting the strongest activity. At the same time, the sample with a 15% mulberry addition exhibits intermediate activity, but significantly higher than the snack without additives, while the results for all extracts are comparable to or only slightly weaker than those obtained for GA (Table 6). The free-radical-scavenging activity has been obtained for the highest concentration of extracts (50 µL of extract in 250 µL of analyzed sample what corresponds to 40 mg/mL of sample).

The results of antioxidant activity assays for samples with and without mulberry, as well as for mulberry alone, obtained using various methods (FRAP, CUPRAC, Fe^2+^ chelation, DPPH), paint a consistent picture: the addition of mulberry significantly improves the product’s antioxidant potential, with mulberry fruit extract remaining the strongest antioxidant in all tests. At the same time, the methods differ in their sensitivity to specific mechanisms of action and “rank” the tested samples slightly differently, which has a direct impact on the interpretation of the results and the assessment of correlations between them.

### 2.4. Determination of Acetylcholinesterase (AChE) Inhibition Using the Ellman Method

Determination of AChE inhibition using the Ellman method is one of the most commonly used techniques in enzyme activity research, particularly in pharmaceutical and toxicological contexts and in analyses related to neurodegenerative diseases [16]. The results indicate that mulberry fruit and the enriched snack possess in vitro AChE inhibitory activity under the tested conditions, while the sample without the additive shows a significantly weaker effect. At the same time, the obtained values are comparable to the inhibition observed in the galantamine control system, which requires cautious biological interpretation. However, further mechanistic and bioavailability studies are needed before any neuroprotective relevance can be inferred (Table 7).

A comparison of 9E and 3E indicates that the mulberry addition significantly increases the percentage of AChE inhibition (approx. 79% vs. approx. 60%), which may be attributed to the presence of polyphenols and other secondary metabolites of mulberry, for which potential neuroprotective properties and the ability to modulate the activity of enzymes involved in neurotransmission have been described in the literature. The similar level of inhibition observed for the raw material and the mulberry snack suggests that the processing (extrusion, drying) does not lead to a complete loss of the compounds responsible for the effect on AChE—their activity is still “transferred” to the final product. At the same time, however, the difference between 9E/13E and galantamine falls within the standard deviation, making it difficult to unequivocally attribute the effect solely to the bioactive compounds of mulberry. All tested samples showed lower activity in comparison to galantamine used as reference compound.

### 2.5. Digestibility of the Snack’s Polyphenols Using a Two-Stage In Vitro Human Digestion Model

Table 8 shows the TPC after the first (gastric) and second (duodenal) phases of in vitro digestion. In addition to absolute TPC values, percentage retention was calculated for each digestion step. Snacks enriched with mulberry (9E) retained approximately 69% of their initial TPC after the gastric phase and about 33% after the intestinal phase, whereas the control snack (3E) retained only 55% and 20%, respectively. White mulberry fruit (13E) showed a similar pattern, with about 70% retention after the gastric phase and approximately 33% after the intestinal phase.

The results of in vitro digestion show a consistent pattern: the total polyphenol content (TPC) and the concentrations of individual phenolic compounds decrease significantly after the gastric phase and the subsequent duodenal phase, both in the mulberry-free snacks, the mulberry-containing snacks and in the mulberry itself, with the samples containing mulberry retaining a relatively larger portion of their polyphenol fraction (Table 9, Table 10 and Table 11).

Results in Table 9, Table 10 and Table 11 indicated that in vitro digestion led to a substantial decline in both total and individual polyphenols, with the most pronounced losses occurring after the intestinal phase. In snacks without mulberry, most compounds retained only about 20–35% of their initial levels after the gastric step and often less than 20% after the intestinal step, indicating relatively low stability of cereal-derived phenolics during digestion. In snacks with 15% mulberry, a similar downward trend was observed, but several chlorogenic acids and flavonol glycosides showed higher gastric retention (often around 30–50%), suggesting that the mulberry matrix provides partial protection to these compounds. Mulberry fruit itself exhibited the highest absolute contents and generally comparable or slightly higher retention percentages than the snacks, with many key phenolics maintaining roughly 25–55% of their initial levels after the gastric phase and around 5–20% after the intestinal phase.

The polyphenolic fraction in mulberries and in snacks containing them is better “protected”—both by the original fruit matrix and by the structure of the extruded product—which results in a higher percentage of compounds remaining intact after both stages of digestion. This can be explained, among other things, by the higher proportion of compounds with more stable structures (chlorogenic acids, flavonol glycosides) and their interactions with polysaccharides and proteins, which limit immediate degradation in the digestive environment.

The results of the in vitro digestion experiments showed that, despite a marked decrease in total and individual polyphenols after the gastric and intestinal phases, snacks enriched with mulberry retained a quantifiable fraction of their polyphenolic constituents in the simulated intestinal environment. This indicates that a portion of these compounds remains stable enough to persist in the later stages of the in vitro gastrointestinal model. However, it should be emphasized that such findings relate solely to the behavior of polyphenols under simulated digestive conditions and do not provide direct information on their absorption, bioavailability, or physiological effects in vivo.

### 2.6. PCA Results

#### 2.6.1. PCA for Total Polyphenolic Content (TPC) and IC_50_ for DPPH

**Figure 1:**
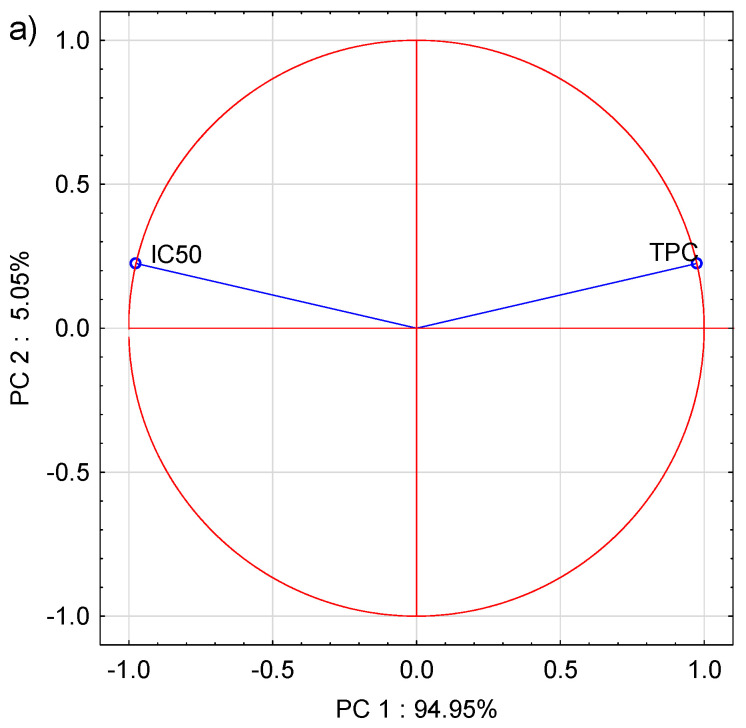

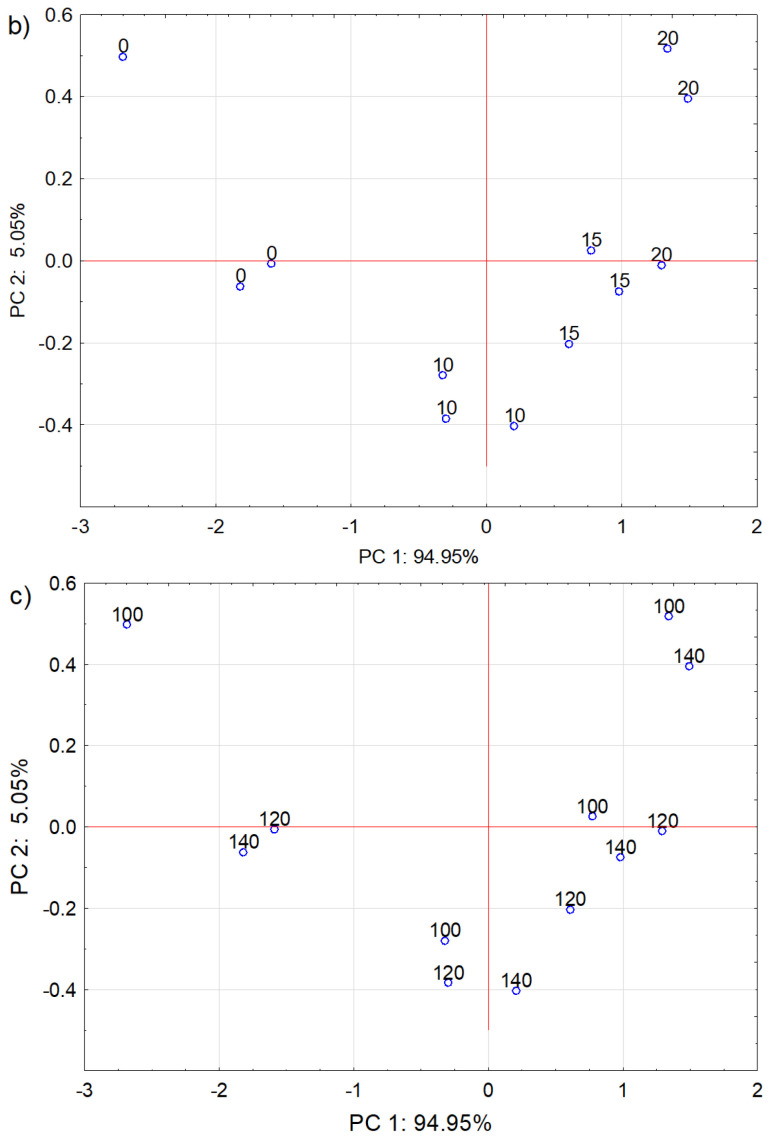
(**a**) Projection of variables: total polyphenolic content (TPC) and IC_50_ for DPPH on the PC1 and PC2 loadings plot. (**b**) Projection of mulberry content on the PC1 and PC2 scores plot. (**c**) Projection of production temperature on the PC1 and PC2 scores plot.

Figure 1 presents the results of principal component analysis for total polyphenolic content (TPC) and IC_50_ for DPPH. The two principal components describe the variability of the studied system to 100%. They indicate that the addition of mulberry has a significant effect on the properties of the obtained extrudate, PC1 = 94.95% (Figure 1a,b), related to total polyphenolic content (TPC) and IC_50_. The amount of additive used has a very small effect, described by the second principal component, PC2 = 5.05%. Process temperature has no effect (Figure 1a,c).

A positive correlation was found between IC_50_ and the product without mulberry additive (Figure 1a,b). The same correlation was obtained between TPC and the use of mulberry additive, with the higher the mulberry additive, the greater the correlation (Figure 1a,b).

#### 2.6.2. PCA for Chromatographic Analysis of Active Compounds

**Figure 2:**
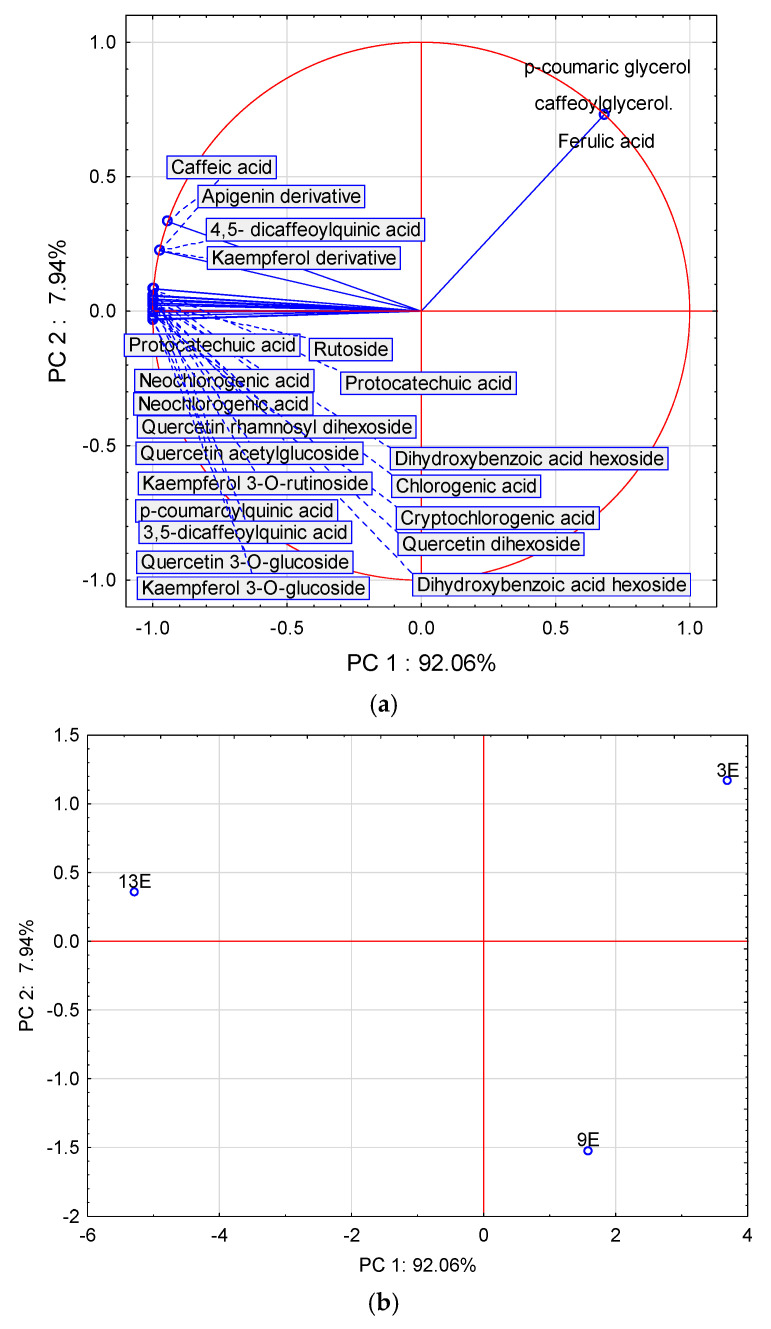
(**a**) Projection of variables: chromatographic analysis of active compounds on the PC1 and PC2 loadings plot. (**b**) Projection of mulberry content (9E, 13E) and no addition (3E) on the PC1 and PC2 scores plot.

Figure 2 presents the results of principal component analysis for chromatographic analysis of active compounds. The two principal components describe the variability of the studied system to 100%.

The first principal component, PC1, accounts for 92.06% of the system’s variability, and the second, PC2, for 7.94%. Active compounds correlated with 3E: p-coumaric glycerol, ferulic acid and caffeoylglycerol. The remaining active compounds are strongly correlated with E4 (Figure 2a,b). PC1 describes the differences between 3E, 9E and 13E (Figure 2b).

#### 2.6.3. PCA for Assessment of Antioxidant Properties and AChE Inhibition

**Figure 3:**
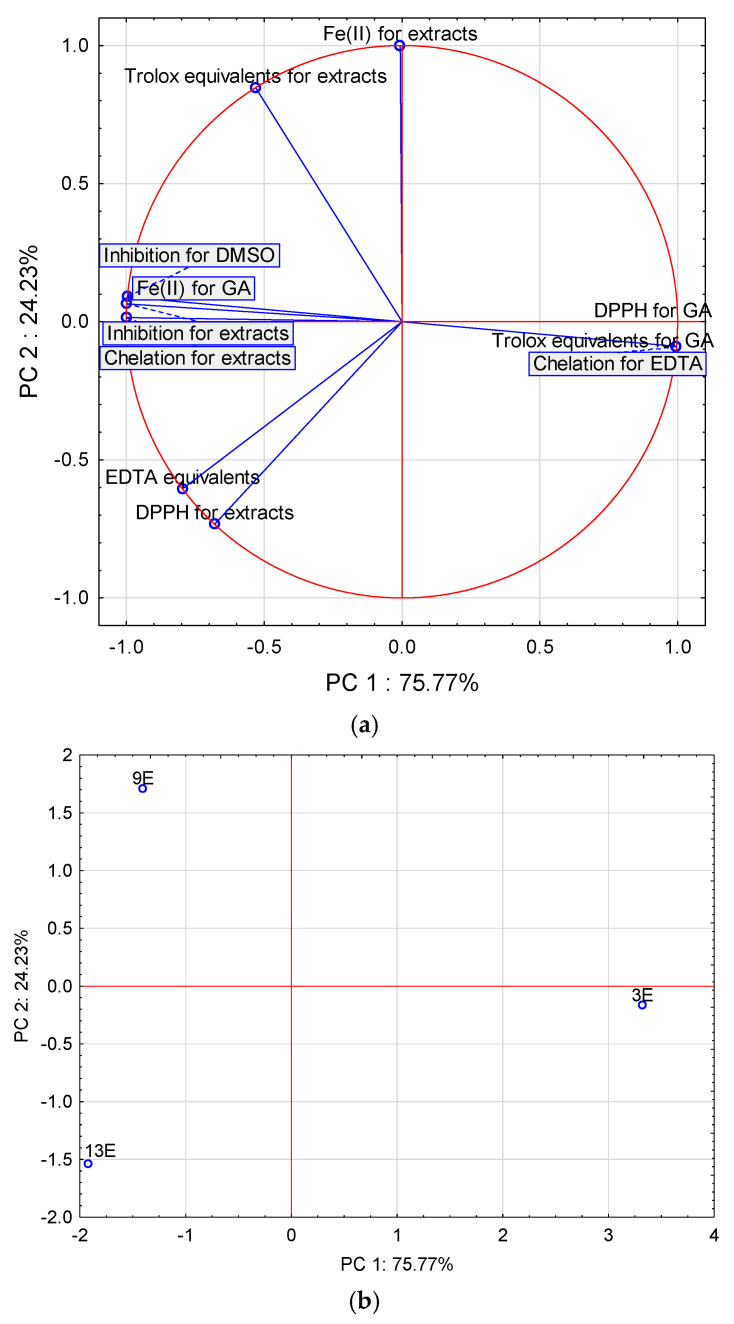
(**a**) Projection of variables: assessment of antioxidant properties and acetylcholinesterase on the PC1 and PC2 loadings plot. (**b**) Projection of mulberry content (9E, 13E) and no addition (3E) on the PC1 and PC2 scores plot.

Figure 3 presents the results of PCA for the assessment of antioxidant properties and AChE inhibition. The first two principal components fully describe the variability of the system (Figure 3a,b).

The analysis shows that the principal component contains 75.77% of the differences between 3E, 9E and 13E, while PC2 contains 24.23% of the abbreviations between 9E and 13E and the parameters that regulate this (Figure 3a,b). DPPH for GA, Trolox equivalents for GA, and chelation for EDTA are strongly and deeply correlated with 3E. Trolox equivalents for extracts and Fe (II) equivalents for extracts are strongly and positively correlated with 9E, and EDTA and DPPH equivalents for extracts are strongly and positively correlated with 13E. The remaining procedure parameters show a negative correlation with E8 and no correlation with unspecified correlations with 9E and 13E (Figure 3a,b).

#### 2.6.4. PCA for Digestibility of the Snack’s Polyphenols Using a Two-Stage In Vitro Human Digestion Model

**Figure 4:**
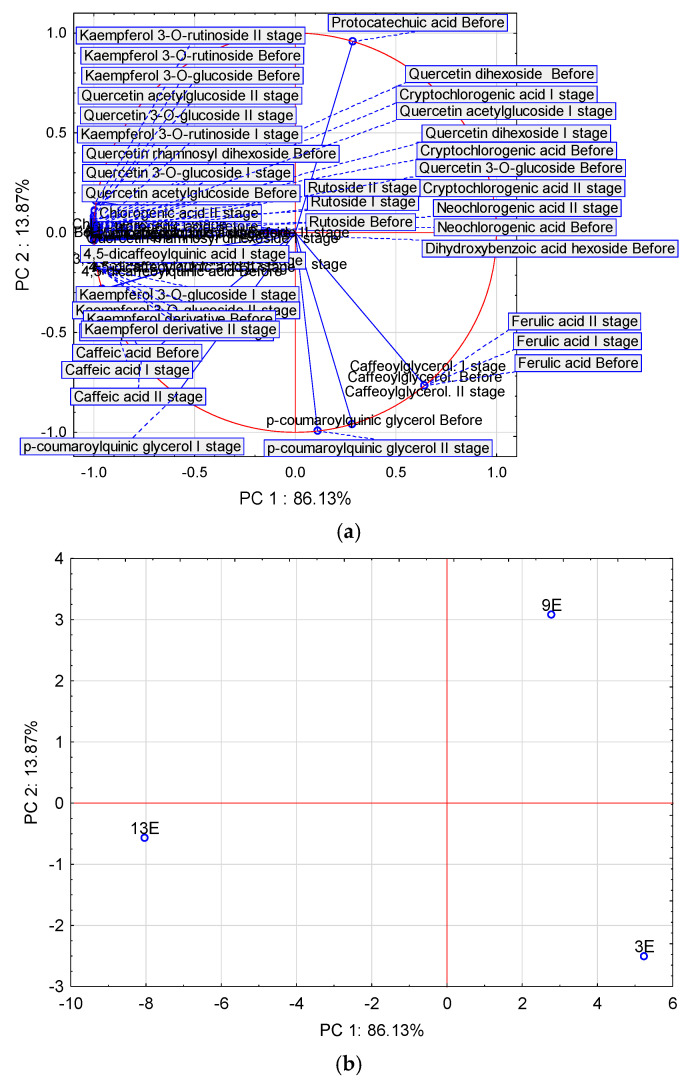
(**a**) Projection of variables: assessment of digestibility of the snack’s polyphenols using a two-stage in vitro human digestion model on the PC1 and PC2 loadings plot. (**b**) Projection of mulberry content (9E, 13E) and no addition (3E) on the PC1 and PC2 scores plot.

Figure 4 shows the results of PCA for the digestibility of the snack’s polyphenols using a two-stage in vitro human digestion model before and after two-stage digestion for mulberry content (9E, 13E) and no addition (3E). The first two components already describe 100% of the system’s variability.

PCA showed (Figure 4a,b) that the parameters Ferulic acid Before, Ferulic acid I stage, Ferulic acid II stage, Caffeoylglycerol Before, Caffeoylglycerol I stage, Caffeoylglycerol II stage, p-coumaroylquinic glycerol Before and p-coumaroylquinic glycerol II stage are strongly and positively correlated with the absence of mulberry 3E addition. Only the parameter Protocatechuic acid Before is strongly and positively correlated with the addition of mulberry 9E. On the other hand, the remaining parameters are strongly and positively correlated with the addition of mulberry 13E.

## 3. Discussion

**Figure 5:**
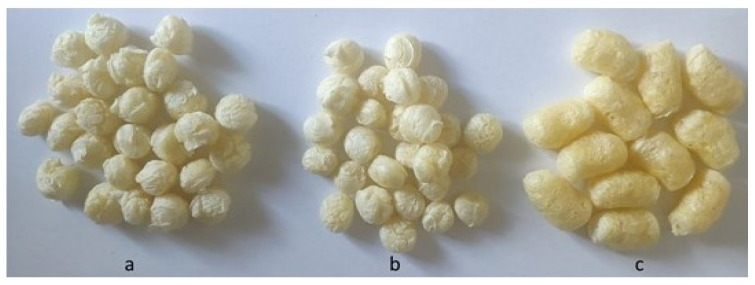
Snacks without mulberry fruit: (**a**) 100 °C, (**b**) 120 °C, (**c**) 140 °C.

**Figure 6:**
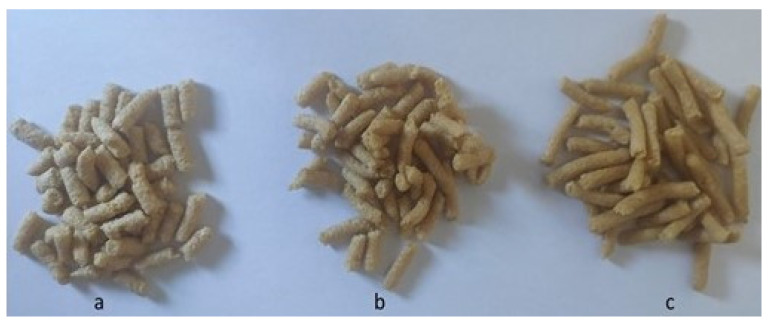
Snacks with 15% mulberry fruit: (**a**) 100 °C, (**b**) 120 °C, (**c**) 140 °C.

The experiment demonstrated that extrusion, a high-temperature, high-pressure production method, did not destroy valuable health-promoting antioxidant components. This is an effective, modern technology that enables the production and marketing of snacks, pasta and porridges. This process involves processing starchy raw materials under high temperature (120–200 °C) and pressure (approx. 20 MPa). Intensive processing, resulting from mechanical shearing, leads to a profound transformation of individual food components. The extrusion temperature, screw speed, moisture content and residence time in the extruder are critical for the nutritional properties, polyphenol content and antioxidant activity of the extrudates [11]. This process can deactivate anti-nutritional factors and enhance the antioxidant properties of foods [17]. Altan and Yağci [18] described the effect of extrusion process variables on the physicochemical, sensory and structural properties of foamed extrudates. The results showed that extrusion of these raw materials led to a significant increase in the levels of low-molecular-weight bioactive compounds they contained. The intensity of these changes depends on both the properties of the raw material (e.g., moisture content) and the processing parameters. A high degree of mixing and homogenization of the raw materials leads to the breaking of diffusion barriers and some chemical bonds. Appropriately selected extrusion conditions can release phenolic acids or flavonoids from the chemical bonds they form with other compounds (e.g., glycosidic bonds). However, this does not result in the deactivation of aglycones. The main factors stimulating the transformation of the feedstock during the extrusion process are related to high temperature and mechanical aspects, namely shear forces, which increase with rising screw speed and temperature [19]. In our case, the most favorable temperature in terms of polyphenol content in snacks with 15% mulberry addition was the highest of those used, namely 140 °C. For snacks without the addition of mulberries, it was 120 °C. We can explain this by the fact that the matrix itself—i.e., the snacks without mulberry differs significantly from the snacks enriched with this fruit (in terms of structure, content and combinations of active ingredients, moisture content and even appearance—see Figure 5 and Figure 6), hence the difference in the optimal extrusion temperature.

The results demonstrated that snacks containing 15% mulberry and dried mulberries have high antioxidant potential, which is consistent with previous studies [14]. However, the results of antioxidant activity measurements vary depending on the method used. One type of antioxidant may perform better in one method and weaker in another—for example, phenols may be more active in the DPPH assay but weaker in the FRAP assay [15]. Furthermore, plants contain many different compounds with antioxidant activity: polyphenols, flavonoids, vitamins (C, E), carotenoids, sulfur compounds, enzymes, etc. Each group has different properties. Some are water-soluble, others fat-soluble. They differ in reaction rate, mechanism of action, and stability over time. In practice, this means that a single plant extract may be classified as a “strong antioxidant” in one method and as a “weak” one in another. Furthermore, processing (e.g., drying, cooking, fermentation) and the production method (and its parameters) can degrade or increase the availability of certain antioxidants. Synergistic and antagonistic effects between the compounds present in the sample are also important [20].

In the initial phase, snacks enriched with 0, 10, 15 and 20% mulberry fruit were tested, extruded at temperatures of 100, 120 and 140 °C. Snacks with a 15% mulberry fruit addition made at 140 °C were selected for further testing. This level represents the “golden mean”: a 15% mulberry addition gives the product functional properties, and the snacks enriched with it exhibited the favorable color and flavor compared to the 20% variants. According to the obtained values, the TPC in snacks with a 15% mulberry content is approximately 3.5 times higher than in the control sample, while dried mulberry is characterized by a polyphenol content approximately five times higher than the extruded product. This pattern is consistent with the literature, in which the functional ingredient generally contains higher concentrations of phenolic compounds than the finished snacks, and an increase in the proportion of the plant component results in a significant increase in the product’s TPC [3,21].

UHPLC analysis showed that the mulberry-enriched snack contains the same groups of compounds as the fruit itself. It can therefore be concluded that these groups of compounds are responsible for the mulberry’s primary antioxidant and health-promoting potential. Their presence in the snack in significant amounts (though lower than in the raw material) is a key argument for treating the product as a functional food. In addition to their strong antioxidant activity, chlorogenic acids influence glucose and lipid metabolism, while quercetin glycosides exhibit, among other effects, anti-inflammatory, antioxidant and potentially cardioprotective properties [22]. A comparison of the concentrations of individual compounds in 9E and 13E shows that the levels in the snack are consistently lower, typically by a factor of 4–6. The extent of the concentration reduction is very similar for most compounds, suggesting that the main cause is the dilution effect (15% mulberry content in the raw material mixture) and the averaging of the content across the entire product volume, rather than the selective degradation of specific classes of polyphenols. In other words, mulberry “contributes” its profile to the snack, while the corn matrix primarily serves as a carrier, diluting the content per unit of volume/mass of the extract. The slight differences in the degree of “decline” between the groups can be explained by variations in the stability of the compounds during mulberry drying, grinding and extrusion, as well as possible interactions with the starch and protein matrix (e.g., complex formation with amylose) [23,24].

Chen et al. [5] described the potential of mulberry as a functional food ingredient. The fruits of *Morus alba* L. are rich in bioactive compounds. These compounds exhibit strong biological activity, demonstrating excellent pharmacological effects against various diseases. The conducted studies unequivocally confirmed the high antioxidant potential of the analyzed plant material. The high content of polyphenols contributed to significant antioxidant activity, as confirmed by several complementary tests. The results obtained indicate significant potential for using the tested raw materials as natural functional ingredients in the production of health-promoting foods. Their use may contribute to increasing the nutritional and biological value of food, while supporting the body in combating oxidative stress. Furthermore, the natural origin of these additives may address the growing consumer demand for products with clean ingredients and health-promoting effects.

An interesting point is that it is not the mulberry fruit extract, but the extract from the sample containing 15% mulberry that shows the highest values in the FRAP analysis. This can be explained by the fact that in the 15% mulberry snack, mulberry polyphenols coexist with components of the corn base and Maillard reaction products formed during extrusion (melanoidins), which themselves exhibit reducing activity. The sum of these components may result in a higher FRAP signal than the fruit alone. Extrusion and prior grinding may increase the extractability of some mulberry polyphenols (rupture of cell walls, alteration of the structure of complexes with proteins/polysaccharides), which in the FRAP assay translates to a greater number of compounds “visible” as Fe^3+^ reducers. Although, on a dry weight basis, mulberry has a higher total polyphenol content than the 15% snack, under specific extraction and FRAP test conditions, some compounds from the raw material may be less extractable or less reactive in this system than the mixture of compounds present in the extruded product [25]. Furthermore, FRAP is particularly sensitive to the presence of specific structures (e.g., ortho-dihydroxyphenols), which may be present in both mulberry polyphenols and starch and protein processing products [15,25]. Conversely, the higher 3E activity compared to 13E indicates that the corn matrix and its thermal processing products make a significant contribution to the results obtained by the FRAP method—despite the very low content of typical polyphenols, other reducing agents (e.g., sulfur compounds) may be present.

It is worth noting that the FRAP method should be performed in conjunction with other tests (CUPRAC, DPPH, Fe^2+^ chelation), as only this approach allows for a more complete antioxidant profile of the tested samples and confirms the validity of using mulberry fruit as a functional additive in snacks. In the CUPRAC method, as in the FRAP method, the highest values were obtained for mulberry samples. As before, this can be explained by several overlapping effects, including synergy with the starch matrix and Maillard products. Furthermore, thermomechanical processing breaks down cell wall structures and polyphenol–protein/polysaccharide complexes, which increases the amount of compounds “visible” in the CUPRAC reaction [26]. Thus, using the same extraction protocol, different fractions of compounds can be recovered from the 13E and 9E—some of the mulberry polyphenols may be more effectively extracted from the snack than from the fruit itself [26]. Both methods, however, “rank” the 9E:13E ratio slightly differently, which results from differences in mechanisms. This once again confirms that mulberry contributes a significant fraction of compounds to the product that act through multiple mechanisms: as electron donors, radical scavengers and potential metal ion chelators. The difference compared to FRAP may stem from the different nature of this test. CUPRAC operates at a milder pH and is more “universal” with respect to various classes of antioxidants (phenols, vitamin C, thiols, etc.), as well as less susceptible to protein interference than FRAP [20]. Some compounds present in mulberry (e.g., selected flavonoid glycosides) may exhibit greater reactivity toward Cu^2+^ than Fe^3+^, which at higher concentrations (e.g., 30% mulberry) translates to a proportional increase in CUPRAC activity; whereas at 15%, the addition is already “sufficient” for FRAP [15]. In practice, this means that FRAP and CUPRAC show a consistent trend (increased activity with increasing mulberry content) and a high correlation with TPC, but FRAP more clearly indicates the “optimality” of the 15% addition, while CUPRAC reacts more dynamically to a further increase in mulberry content up to 30%.

In a test involving the chelation of Fe^2+^ ions, the mulberry extract achieved 100% chelation at the highest EDTA equivalent, which indicates that it contains high concentrations of compounds with the appropriate geometry and number of functional groups (orthodihydroxyphenols, carboxyl groups), capable of forming stable complexes with iron ions. This is consistent with the UHPLC analysis of mulberry fruit, where chlorogenic acids and their derivatives, as well as flavonol glycosides, known for their good metal-chelating properties, predominate. The enriched snack exhibits very high chelation (approx. 97%), although the EDTA equivalent is significantly lower than in 13E. This indicates that the mulberry fraction contributes significant Fe^2+^-binding capacity to the product; however, the total chelating “power” per unit volume of extract is diluted by the starch matrix and the lower proportion of plant material (15%). The result for 9E is nevertheless significantly higher than for the baseline sample, confirming the effectiveness of enrichment in the context of this antioxidant mechanism. The chelation of Fe^2+^ ions is one of the key mechanisms for limiting the formation of free radicals in Fenton reactions. Compounds capable of binding Fe^2+^ “remove” it from the cycle of reactions that generate hydroxyl radicals, which are among the most reactive ROS in biological systems. The results obtained suggest that mulberry extract can act as an effective “trap” for iron ions.

The DPPH assay primarily assesses the ability to transfer hydrogen atoms or electrons to the stable DPPH radical. The very high DPPH-scavenging percentage by 13E (exceeding the result obtained for GA) confirms that mulberry fruit is a rich source of compounds capable of rapidly donating hydrogen atoms/electrons to the stable DPPH radical. This is fully consistent with the profile of the polyphenolic fraction of mulberry fruit and with the high TPC. From a practical standpoint, this means that even a small amount of mulberry extract can have a strong free radical “scavenging” effect in the DPPH model system. The 9E activity is lower than 13E, but significantly higher than for the product without the additive, and similar to the GA activity. The result for 9E, which is close to that of GA, is significant in terms of interpretation: a processed product that naturally achieves activity comparable to a 1 mM solution of a strong reference antioxidant is of considerable importance as a dietary component with antioxidant potential [24]. The lowest DPPH-scavenging percentage in 3E reaffirms that the corn matrix itself contributes moderate antioxidant potential.

The FRAP, CUPRAC, Fe^2+^ chelation and DPPH methods provide a consistent picture: the addition of mulberry enhances the antioxidant potential of the snacks, and mulberry extract remains the strongest antioxidant in all the tests used. DPPH particularly highlights the higher antioxidant potential of the raw material compared to the enriched product, whereas, for example, FRAP or CUPRAC sometimes favor the mulberry snack. Observed correlations between the methods and between them and TPC indicate that the addition of mulberry to snacks is justified from the perspective of increasing the product’s antioxidant potential, but at the same time underscore the need to use a set of complementary methods rather than a single test [27]. The FRAP, CUPRAC, Fe^2+^ chelation and DPPH assays together provide a coherent picture in which the addition of mulberry enhances the antioxidant potential of the snacks, with mulberry extract generally remaining the strongest antioxidant in the test panel. At the same time, these methods differ in their primary mechanisms and therefore rank the samples somewhat differently: FRAP and CUPRAC are predominantly electron transfer based metal-reducing assays (Fe^3+^/Fe^2+^ and Cu^2+^/Cu^+^, respectively), the ferrozine test evaluates the ability of compounds to chelate Fe^2+^ ions, and DPPH primarily reflects radical-scavenging capacity via hydrogen atom or single-electron transfer. Such mechanistic differences, well-documented in the literature for plant extracts and food matrices, explain why a given sample may appear relatively stronger in one assay and weaker in another, and underline the need to use a set of complementary methods rather than a single test when characterizing antioxidant activity.

AChE is a key enzyme responsible for the breakdown of acetylcholine at synapses, and its pharmacological inhibition is a standard strategy in, among other things, the symptomatic treatment of Alzheimer’s disease. The results showed high in vitro AChE inhibitory activity mulberry extract and products. Combined with the previously demonstrated strong antioxidant properties of mulberry and snacks containing it, one can speculate about the synergy between antioxidant activity and the modulation of AChE activity, which aligns with the concept of functional foods supporting cognitive function. The results consistently show that the addition of mulberry modifies the samples’ activity against AChE—the snack containing 15% mulberry and the fruit itself exhibit higher inhibitory potential than the control sample. Combined with previous UHPLC results and antioxidant tests, this paints a consistent picture of a product with functional potential [28]. The results suggest in vitro potential anticholinesterase activity of mulberry and the product containing it but require further clarification in subsequent studies.

The results of the two-stage in vitro digestion clearly show that the total polyphenol content and the concentrations of individual phenolic compounds decrease significantly after the gastric and intestinal phases in all tested samples, with mulberry-enriched snacks and mulberry fruit retaining a relatively higher percentage of the phenolic fraction than the control snack. After the gastric phase, the decrease in TPC reaches approx. 45% in the sample without mulberry, while in the enriched snacks and in the mulberry fruit it is approx. 30%, and after the intestinal phase, TPC settles at approx. 20% of the initial value in the control sample and approx. 30% in the mulberry-containing samples. These values fall within the range reported for other plant products and extruded snacks, in which 20–40% of the initial polyphenol content or its antioxidant equivalents are typically recovered after simulated digestion. It is worth noting that the observed differences between the control sample and the samples containing mulberry reflect both the distinct profile of phenolic compounds and the role of the food matrix in the protection and release of polyphenols. The mulberry-free snacks contain mainly phenolic acids with a relatively simple structure (including chlorogenic acid, ferulic acid), which benefit to a lesser extent from the “buffering” effect of dietary fiber and other components of the plant matrix, which may promote their faster degradation or transformation under conditions of low pH and the action of pepsin. In contrast, in samples containing mulberries, the primary structure of the fruit (cell walls–polyphenol–polysaccharides–proteins) and the presence of numerous chlorogenic acids and flavonol glycosides, which are relatively more stable under gastric conditions, as confirmed by both our results and previous reports on the resistance of many phenolic acids and flavonoids to the acidic gastric environment [3,29].

Qualitative analysis of the profiles of individual compounds at successive stages of digestion indicates a clearly differentiated sensitivity of individual classes of polyphenols. In mulberry snacks and in mulberries themselves, compounds such as dihydroxybenzoic acid hexoside and protocatechuic acid, as well as some of the more labile caffeic and apigenin derivatives, which is consistent with observations by other authors that some hydroxybenzoic acids and anthocyanins undergo significant degradation or transformation under the influence of low pH and elevated temperature. At the same time, chlorogenic acids and their isomers, p-coumaroylquinic acid and ester derivatives of caffeic acid remain detectable after both the gastric and intestinal phases, although their concentrations decrease steadily, indicating the relative stability of these structures as they pass through the upper gastrointestinal tract [30]. A similar stability pattern applies to flavonol glycosides, such as rutin, quercetin 3-O-glucoside, quercetin dihexoside, quercetin acetylglucoside and kaempferol. In mulberry samples, these compounds are present in high concentrations prior to digestion; after the gastric phase, their content decreases significantly, but they remain detectable even after the intestinal phase, suggesting their partial resistance to conditions in the duodenum and the possibility of further transformations in the small and large intestines. The literature describes that quercetin and kaempferol glycosides are only minimally absorbed in the upper intestine and largely reach the large intestine, where they undergo intensive metabolism involving the microbiota, leading to the formation of simpler phenolic acids that still exhibit biological activity [30].

The 60–80% decrease in TPC observed in our model following the duodenal phase should therefore not be interpreted solely as a loss of biological potential, but rather as the result of chemical degradation, isomerization, hydrolysis of phenolic glycosides and the conversion of some compounds into forms that are not detected as “polyphenols” in the classic Folin–Ciocalteu test. Numerous studies have shown that native polyphenols are only partially absorbed in the small intestine, and a significant fraction reaches the large intestine, where, under the influence of microorganisms, they are converted into low-molecular-weight metabolites (including derivatives of benzoic, phenylpropionic and phenylcarboxylic acids), which play an important role in influencing lipid metabolism, insulin sensitivity, inflammation and endothelial function [31,32]. The results of our experiment, in which residual amounts of chlorogenic acids and flavonol glycosides are still detectable after intestinal digestion, and TPC in mulberry samples remains significantly higher than in the control, are thus consistent with the concept that mulberry fruits and snacks containing them can deliver a significant number of substrates for the gut microbiota to the distal sections of the gastrointestinal tract.

Another key aspect of this interpretation is the role of extrusion and the structure of the starch matrix in determining the bioaccessibility of mulberry polyphenols. As studies on cereals, fruits and extruded products show, intensive thermal–mechanical processing can, on the one hand, cause the degradation of some polyphenols, and on the other, increase their release from complexes with fiber and proteins and improve their accessibility to digestive enzymes, which ultimately leads to an increase in the bioaccessible fraction after simulated digestion [11]. In our case, the polyphenolic fraction in mulberries and in the snacks containing them appears to be more “protected” than in the control sample—after both digestion stages, samples 9E and 13E retain significantly higher TPC and a broader spectrum of compounds than 3E, which can be explained by both a more stable compound profile (dominance of chlorogenic acids and flavonol glycosides) and interactions with polysaccharides and proteins that limit immediate degradation in the digestive environment. At the same time, it should be noted that the matrix of the extruded snack may modify the rate and site of phenolic compound release in the gastrointestinal tract. Preliminary grinding and gelatinization of starch promote more uniform contact with digestive juices and may facilitate the transfer of some polyphenols to micelles and the soluble fraction in the intestinal phase, which in the literature is associated with greater bioaccessibility and potential bioavailability of phenolic acids after extrusion [33]. In this context, the fact that the quality profile of the compounds present in the 15% mulberry snacks after both stages of digestion is very similar to that of mulberry, albeit at lower concentrations, should be viewed as an advantage: the extruded product delivers key phenolic structures to the intestinal environment in a form that is more technologically and sensorially convenient, while preserving their ability to potentially interact with epithelial cells and the microbiota.

It can therefore be concluded that the inclusion of *Morus alba* L. fruit in the gluten-free snack matrix not only increases its in vitro antioxidant potential but also ensures that a significant portion of this fraction remains available after passing through the upper gastrointestinal tract and may potentially exert a biological effect in vivo.

## 4. Materials and Methods

### 4.1. Preparation of Snacks

Corn-based formulations were prepared by mixing corn grits with dried white mulberry fruit at four levels: 0, 10, 15 and 20% mulberry (*w*/*w*, on a dry matter basis). Dried mulberry fruits were first ground in a laboratory mill and sieved to obtain a powder with particle size below 500 μm, while the corn grits had a particle size in the range of approximately 500–1000 μm. Before extrusion, the mixtures were thoroughly homogenized and their moisture content was adjusted to 12% (*w*/*w*) by spraying with a calculated amount of distilled water and mixing, followed by equilibration in sealed polyethylene bags at 4 °C for at least 12 h. The mulberry fruit used in the formulations had been dried in a forced-air dryer at 60 °C to constant weight and then cooled to room temperature prior to milling. Processing was carried out at 100, 120 and 140 °C using a multi-screw extruder (extrusion parameters: screw speed 600 rpm, feed rate 12 kg/h), and the resulting snacks Figure 5 and Figure 6) were milled before extraction and analysis. All four formulations (0, 10, 15 and 20% mulberry) were extruded under identical screw speed and feed rate settings to allow for a direct comparison of the effect of mulberry addition on the properties of the snacks.

The results identify the 15% mulberry snacks produced at 140 °C as the best preliminary sample and this formulation was therefore used in the later expanded assays together with the 0% (140 °C) control and mulberry fruit alone. This design allowed for direct comparison of the baseline cereal matrix, the optimized enriched snack and the raw plant material [34].

Sample labels: 9E—snack with 15% mulberry fruit added, 13E—mulberry fruit, 3E—snack without additives.

### 4.2. Preparation of Polyphenol Extracts Using the ASE Method

Polyphenol extracts were prepared using accelerated solvent extraction [34], which is described in the methodological attachment as a high-temperature, high-pressure extraction technique suitable for phenolic compounds. The ASE process was carried out under the following constant conditions: ethanol as the solvent, pressure—1500 psi; temperature—65 °C; furnace preheating—5 min; chamber heating—5 min; static extraction time—10 min; volume of pure solvent rinse—60%; purification time—60 s. The use of ASE helped standardize extraction before DPPH, FRAP, CUPRAC, chelation, AChE and UHPLC testing.

### 4.3. Determination of Total Polyphenol Content

Total polyphenols were measured with the Folin–Ciocalteu method, using gallic acid as the calibration standard [3]. Absorbance was read at 765 nm in this procedure and the results were expressed as gallic acid equivalents. This assay was used to compare the four mulberry levels at all three temperatures and to identify the best preliminary formulation. The 15% mulberry, 140 °C sample gave the best overall polyphenol result and was therefore selected for extended tests. To ensure reliability, the measurements were repeated four times.

### 4.4. Determination of IC_50_

IC_50_ was determined from the DPPH assay as the concentration of extract required to reduce the initial DPPH radical concentration by 50% [34].

For this purpose, a series of extract dilutions was prepared, and different volumes of each extract were added to the reaction mixture to obtain final sample concentrations of 40, 20, 12.5 and 6.25 mg/mL. IC_50_ values (mg/mL of reaction mixture) were calculated from the corresponding dose–response curves (DPPH scavenging vs. sample concentration). To ensure reliability, the measurements were repeated four times. A lower IC_50_ indicates stronger antioxidant activity, and this parameter was therefore used to directly compare the antioxidant potential of all tested samples, including the pure mulberry fruit extract and the various snack formulations.

### 4.5. UHPLC Analysis

UHPLC analysis was performed for the mulberry fruits, 15% mulberry snacks and the plain control snacks, using the ASE-prepared extracts [3]. Analyses were performed using an Agilent 1290 Infinity II UHPLC system equipped with a diode array detector (DAD) and an Agilent 6224 ESI/TOF mass spectrometer (Agilent Technologies, Santa Clara, CA, USA), coupled with a Kinetex C18 reversed-phase column (100 Å, 150 × 2.1 mm, 1.7 µm particle size; Phenomenex, Torrance, CA, USA). Column temperature was set at 30 °C and the flow rate at 0.2 mL/min. The mobile phase consisted of water with 0.05% formic acid (solvent A) and acetonitrile with 0.05% formic acid (solvent B). Gradient elution was applied as follows: 0–8 min, 98–93% A; 8–15 min, 93–88% A; 15–29 min, 88–85% A; 29–40 min, 85–80% A; 40–80 min, 80–55% A. UV–Vis data were recorded in the wavelength range of 200–600 nm. Mass spectrometry conditions included a drying gas temperature of 325 °C, gas flow rate of 8 L/min, nebulizer pressure of 30 psi, capillary voltage of 3500 V, fragmentor voltages of 160, 200 and 320 V, and skimmer voltage of 65 V. Ions were detected in the range of 100–1200 *m*/*z* in negative ion mode. Compounds marked with an asterisk in Table 2 and Table 3 were identified by comparison with authentic standards (Sigma-Aldrich, St. Louis, MO, USA) (confirmation level 1). The remaining compounds were tentatively identified based on MS data and literature comparison (confirmation level 2).

Compounds were quantified based on DAD detection. Method validation parameters are provided in Supplementary Table S1. In cases where authentic standards were not available, calibration data of structurally related compounds with the corresponding aglycone were used for quantification.

### 4.6. Determination of Iron Ion Reducing Capacity—FRAP Method

FRAP was used to assess the ability of extracts to reduce Fe^3+^ to Fe^2+^ through electron donation. In this method, a Fe-TPTZ complex is formed. The spectrophotometric measurement is performed at a wavelength of 593 nm [35] with minor modifications. The concentrations of the analyzed extracts were identical to those used in the DPPH assay. The FRAP reagents were freshly prepared before each experiment by mixing 0.3M acetate buffer (pH 3.6), 0.01 M TPTZ dissolved in 0.04 M HCl and 0.02 M FeCl_3_ × 6H_2_O in a volume ratio of 10:1:1 (*v*/*v*/*v*). The reagent was protected from light until use. For the assay, 50 µL of each sample solution was combined with 200 µL of FRAP reagent in a microplate well. The mixtures were vortexed and incubated at 37 °C away from light. Absorbance was subsequently recorded at 593 nm every 5 min for 30 min using a SPECTROstar Nano microplate reader (Ortenberg, Germany). A blank sample consisted of the FRAP working solution mixed with methanol instead of the extract, whereas gallic acid was served as the positive control. Reducing power was quantified using calibration curved prepared with aqueous FeSO_4_ solution and expressed as Fe^2+^ equivalents (µg/mL) using appropriate calibration curve. To ensure reliability, the measurements were repeated four times.

### 4.7. Determination of Cu^2+^ Ion Reducing Capacity—CUPRAC Method

CUPRAC measured the reducing power of extracts toward copper ions in the presence of neocuproine. In this method, reduction of Cu^2+^ to Cu^+^ produces a colored complex whose absorbance is measured spectrophotometrically [35]. The concentrations of the tested extracts were the same as those in previous assays, namely: 40, 20, 10 and 5 mg/mL. Briefly, 50 µ of each extract solution was mixed with CuCl_2_ (10mM), neocuproine (7.5 mM) and ammonium acetate buffer (1 M, pH 7.0) in a microplate well. Subsequently, 50 µL of distilled water was added to obtain a final reaction volume of 250 µL. The reaction mixtures were incubated in room temperature in the dark by 10 min. Thereafter, absorbance was measured at 450 nm. Gallic acid was served as positive control. Antioxidant capacity was calculated from calibration curve and expressed as Trolox equivalents (µmol TE/g dry matter). All measurements were performed in quadruplicate.

### 4.8. Determination of Iron (II) Ion Chelating Capacity

Iron chelation was measured using ferrozine, which forms a colored complex with Fe^2+^ unless chelating agents bind the metal first [35]. The concentrations of the tested extracts were the same as those in the previous assays. Briefly, 50 µL of each extract solution was mixed with a 2 mM Fe (II) solution and incubated in the dark for 10 min. Subsequently, 5 mM ferrozine solution was added, and the reaction mixtures were incubated for an additional 10 min in the absence of light. The volume of each sample then adjusted to 250 µL by the addition of MeOH. Absorbance was measured at 562 nm using a microplate reader. EDTA was employed as the reference chelator. All measurements were performed in quadruplicate. The results are presented as percentage chelation of Fe (II) and EDTA equivalents (µg/mL).

### 4.9. Determination of DPPH-Free-Radical-Scavenging Activity

The free-radical-scavenging capacity of the samples was determined using DPPH assay. The procedure was based on the methods described by [3] with minor modifications. A 200 µM DPPH solution was prepared by dissolving the appropriate amount of DPPH reagent in absolute methanol. The tested extracts were diluted in methanol to obtain concentrations of 40, 20, 10 and 5 mg/mL. Subsequently, 200 µL of the DPPH solution was added to each well of a microplate containing the extract samples, methanol as the blank control, 1 mM gallic acid as a positive control and EtOH as a negative control. Absorbance was measured at 517 nm at 5 min intervals over a total incubation period of 30 min. Antioxidant activity was expressed as the percentage of free radical scavenging activity and IC_50_ values [3]. All measurements were performed in quadruplicate.

### 4.10. Determination of AChE Inhibitory Capacity

AChE inhibition was determined using the Ellman method [28], in which enzymatic hydrolysis generates a measurable chromophore unless the extract inhibits the enzyme. The results were interpreted as a potential indicator of neuroprotective relevance. Basis of determination of AChE inhibitory activity was spectrophotometric Ellman assay with slight modifications [28]. The concentrations of the tested extracts were the same as those in previous assays, namely: 40, 20, 10 and 5 mg/mL. Solutions of enzyme AChE (0.25 U/mL) were prepared in phosphate buffer (0.1 M, pH 8). Additional reagents: acetyltiocholine or butyryltiocholine iodide (14 mM) and DTNB (10 mM) were also prepared in phosphate buffer (0.1M, pH 8). The following procedure was used: 50 µL of solution of studied extracts, 20 µL of AChE solution were mixed with 140 µL of phosphate buffer pH 8, 0.1 M in a 96-well plate. After 15 min incubation in 25 °C substrate solution (10 µL) and DTNB (10 µL) were added to the reaction. Absorbance changes were recorded every 5 min to 30 min. As a standard substance galantamine (1 mg/mL) was used. The results were expressed as the % inhibition of enzymes and compared with reference compound. All measurements were performed in quadruplicate.

### 4.11. In Vitro Two-Stage Digestion Model

Dry samples (snacks) were used in the study. The authors employed a static in vitro digestion model comprising two sequential phases (gastric and duodenal) as proposed by the United States Pharmacopeia (2000) based on Seraglio et al., [36] with minor modifications. The gastric phase was initiated by accurately weighing 1.632 g of each sample, which was then homogenized. Subsequently, 5.84 mL of simulated gastric fluid was added, and the mixture was manually stirred for 4 min. This was followed by the addition of 2.32 mL of hydrochloric acid, adjusting the pH to 2.5 ± 0.2, and incubation in a shaking water bath (37 °C, 100 rpm) for 2 h. After incubation, the samples were centrifuged (10 min, 8000 rpm), and the resulting supernatants were collected for analysis and stored at −20 °C for 24 h until use.

The duodenal phase was performed analogously to the gastric phase but using 2.246 g of sample. After incubation (omitting the centrifugation step at this point), 0.09 mL of 1 mol/L sodium bicarbonate was added to each sample to raise the pH to 5.5, followed by the addition of 2.26 mL of simulated duodenal fluid and 1 min of mixing. Subsequently, 0.72 mL of sodium bicarbonate solution was added to adjust the pH to 6.7 ± 0.2. The samples were then incubated for 2 h in a shaking water bath (37 °C, 100 rpm). After this stage, centrifugation was carried out (10 min, 8000 rpm), and the supernatants were collected and stored under the same conditions as those from the gastric phase.

The simulated gastric fluid was prepared by dissolving 0.16 g of pepsin in 0.35 mL of 12 M hydrochloric acid and diluting the mixture to 50 mL with ultrapure water. The simulated intestinal (duodenal) fluid was obtained by dissolving 0.25 g of pancreatin, 0.047 g of sodium glycodeoxycholate, 0.0505 g of sodium taurocholate, and 0.029 g of sodium taurodeoxycholate in 0.25 mL of 0.5 M sodium bicarbonate and then making up the volume to 25 mL with ultrapure water.

### 4.12. Principal Components Analysis

Statistica software (version 13.0, StatSoft Inc., Tulsa, OK, USA) was used for statistical analyses. Data were analyzed via one-way analysis of variance (ANOVA), considering the group effect as the experimental factor. Tukey’s HSD (honestly significant difference) test was employed for post hoc comparisons. Principal component analysis (PCA) was performed at a significance level of α = 0.05. The data matrix used for principal component analysis of the results for total polyphenolic content (TPC) and IC_50_ for DPPH had 12 rows and 4 columns. The data matrix used for principal component analysis of the results for chromatographic analysis of active compounds had 3 rows and 26 columns. The data matrix used for principal component analysis of the results for the assessment of antioxidant properties and AChE inhibition had 3 rows and 12 columns, and the data matrix used for principal component analysis of the results for the digestibility of the snack’s polyphenols using a two-stage in vitro human digestion model had 3 rows and 59 columns. The number of principal components obtained in the analysis was determined based on Cattel’s criterion. The input matrix was automatically rescaled.

## 5. Limitations of the Study

This study has several limitations that should be considered when interpreting the results. First, the biological activity of the samples was evaluated exclusively using in vitro assays, including antioxidant tests, acetylcholinesterase inhibition and a two-stage simulated gastrointestinal digestion model; therefore, these results should not be interpreted as direct evidence of in vivo efficacy, bioavailability, or neuroprotective effects in humans. Second, the digestion model used in this work reflects only the gastric and intestinal phases and does not account for colonic fermentation, epithelial absorption, metabolic transformation, or interactions with the gut microbiota, all of which may substantially influence the final bioaccessibility and biological relevance of polyphenolic compounds. In addition, the AChE inhibition results should be treated as preliminary screening data and require confirmation using a fully standardized assay design with an appropriate positive control and concentration–response analysis. Finally, the present study focused mainly on chemical and functional parameters, whereas broader technological, sensory and storage-related characteristics should be evaluated in future studies to better assess the application potential of white mulberry-enriched gluten-free snacks.

## 6. Conclusions

The addition of white mulberry fruit to extruded corn snacks significantly increases the total polyphenol content and antioxidant activity of the product. UHPLC analysis demonstrated that both mulberry fruit and the enriched snack contain numerous chlorogenic acids and their derivatives, as well as quercetin and kaempferol glycosides, and that the extrusion process does not lead to the complete degradation of these compounds but mainly to their dilution in the starch matrix. A two-stage in vitro digestion model showed a significant decrease in TPC and the concentrations of individual polyphenols after the gastric and intestinal phases; however, samples containing mulberry (dried fruit and snacks with a 15% addition) retained significantly higher levels of polyphenols than the control sample, indicating better bioaccessibility of the phenolic fraction in systems containing mulberry.

The use of a set of complementary methods (FRAP, CUPRAC, DPPH, Fe^2+^ chelation) confirmed that mulberry is the primary source of antioxidant activity in the tested system, and the matrix of the extruded snack may further enhance this effect. Mulberry fruit extract and snacks with a 15% mulberry addition also exhibited in vitro acetylcholinesterase inhibition, significantly higher than the control sample, which, combined with strong antioxidant potential, positions this raw material and product within the concept of functional foods.

The results obtained indicate that *Morus alba* L. fruit can be used as a functional ingredient in gluten-free extruded snacks, enhancing their health benefits by providing phenolic compounds that remain stable during processing and, to some extent, under digestive conditions.

## Figures and Tables

**Table 1:** Total polyphenolic content (TPC, mg GAE/g dry weight) and IC_50_ values determined for samples in snacks enriched with white mulberry (*n* = 3; mean ± SD).

Sample	Mulberry Content (%)	Production Temperature (°C)	TPC	IC_50_ [mg/mL]
1E	0	100	0.155 ± 0.008	0.950 ± 0.023
2E	0	120	0.265 ± 0.001	0.674 ± 0.034
3E	0	140	0.212 ± 0.006	0.704 ± 0.011
4E	10	100	0.450 ± 0.006	0.408 ± 0.014
5E	10	120	0.435 ± 0.003	0.386 ± 0.016
6E	10	140	0.525 ± 0.011	0.296 ± 0.006
7E	15	100	0.717 ± 0.007	0.272 ± 0.004
8E	15	120	0.637 ± 0.014	0.261 ± 0.011
9E	15	140	0.730 ± 0.012	0.219 ± 0.008
10E	20	100	0.907 ± 0.023	0.259 ± 0.002
11E	20	120	0.800 ± 0.017	0.176 ± 0.003
12E	20	140	0.912 ± 0.038	0.212 ± 0.09
13E	mulberry fruit	-	1.925 ± 0.053	0.0926 ± 0.01

**Table 2:** Concentration [µg/mL] of major polyphenolic compounds in extracts from the sample without mulberry addition.

Rt (min)	[*m*/*z*-H]^−^	Mass Error (ppm)	Formula	Compound	Sample 3E
16.21	353.08831 (191, 179)	1.42	C_16_H_18_O_9_	Chlorogenic acid *	0.34 ± 0.03
16.94	179.03546	2.65	C_9_H_8_O_4_	Caffeic acid *	0.13 ± 0.01
18.38	253.07051 (179)	−4.93	C_9_H_14_O_6_	Caffeoyl glycerol *	1.07 ± 0.06
22.58	237.07711 (163)	1.1	C_12_H_14_O_5_	p-coumaric glycerol	2.28 ± 0.10
26.04	193.05112	2.51	C_10_H_10_O_4_	Ferulic acid *	1.30 ± 0.08

* Identification confirmed by standard (level 1).

**Table 3:** Concentration [µg/mL] of major polyphenolic compounds identified in extracts from sample with mulberry addition (9E) and in mulberry (13E).

Rt (min)	[*m*/*z*-H]^−^	Mass Error (ppm)	Formula	Compound	Sample 9E	Sample 13E
7.79	315.07242	0.84	C_13_H_16_O_9_	Dihydroxybenzoic acid hexoside	0.71 ± 0.02	3.45 ± 0.21
8.67	153.01994	3.95	C_7_H_6_O_4_	Protocatechuic acid *	0.24 ± 0.01	1.55 ± 0.11
11.29	353.08821 (191, 179)	1.14	C_16_H_18_O_9_	Neochlorogenic acid *	3.22 ± 0.06	16.8±0.67
15.05	341.08678 (179)	−3.0	C_15_H_18_O_9_	Caffeic acid hexoside	DNQ	1.06 ± 0.09
16.24	353.08867 (191, 179)	2.44	C_16_H_18_O_9_	Chlorogenic acid *	7.29 ± 0.22	39.32 ± 2.11
18.00	625.13863	−3.82	C_27_H_30_O_17_	Quercetin dihexoside	0.54 ± 0.03	2.26 ± 0.13
18.23	771.19640	−3.28	C_33_H_39_O_21_	Quercetin rhamnosyl dihexoside	1.08 ± 0.01	5.19 ± 0.31
18.72	353.08841 (191, 179)	1.71	C_16_H_18_O_9_	Cryptochlorogenic acid *	0.98 ± 0.00	3.91 ± 0.21
20.32	337.09131 (191)	−4.68	C_16_H_18_O_8_	p-coumaroylquinic acid	0.29 ± 0.01	1.09 ± 0.08
24.28	497.1279 (269)	−4.35	C_22_H_26_O_13_	Apigenin derivative	DNQ	0.36 ± 0.02
31.90	609.14844	3.82	C_27_H_30_O_16_	Quercetin 3-O-rutinoside *	3.57 ± 0.15	16.72 ± 1.13
33.41	463.08814	−0.13	C_21_H_20_O_12_	Quercetin 3-O-glucoside *	1.12 ± 0.04	6.19 ± 0.34
37.48	505.09807 (300)	−1.37	C_23_H_22_O_13_	Quercetin acetylglucoside	1.7 ± 0.03	8.62 ± 0.41
37.91	593.14823 (287)	−4.97	C_27_H_30_O_15_	Kaempferol 3-O-rutinoside *	0.89 ± 0.01	3.85 ± 0.21
38.99	515.11847 (353)	−2.0	C_25_H_24_O_12_	3,5-dicaffeoylquinic acid *	0.29 ± 0.01	1.89 ± 0.11
39.16	447.09155	−3.87	C_21_H_20_O_11_	Kaempferol 3-O-glucoside *	0.31 ± 0.00	1.18 ± 0.08
42.77	515.11825 (353)	−2.42	C_25_H_24_O_12_	4,5- dicaffeoylquinic acid *	DNQ	0.78 ± 0.05
43.21	489.10257	−2.61	C_23_H_22_O_12_	Kaempferol derivative	DNQ	1.07 ± 0.07

* Identification confirmed by standard (level 1); DNQ—detected, not quantified.

**Table 4:** Results for FRAP and CUPRAC assays expressed as Fe (II) concentration and Trolox equivalents, respectively (after 15 min of reaction; extracts concentration 40 mg/mL, *n* = 4; ±SD).

Sample	Fe(II) [µg/mL]	µmol TE/g d.m.
3E	15.1 ^a^ ± 0.29	5.23 ^a^ ± 0.23
9E	20.11 ^b^ ± 0.42	15.65 ^b^ ± 0.85
13E	11.52 ^c^ ± 0.18	13.43 ^c^ ± 1.22

Values in the same column marked with different letter differ significantly (*p* < 0.05).

**Table 5:** Fe^2+^ chelating potential and EDTA equivalents [µg/mL] per extract concentration obtained after 15 min of reaction initiation (*n* = 4; ±SD).

Sample	Chelation [%] for Extracts	EDTA Equivalents [µg/mL]
3E	62.60 ^a^ ± 3.74	104.40 ^a^ ± 3.91
9E	96.91 ^b^ ± 4.61	174.50 ^b^ ± 8.05
13E	100.00 ^c^ ± 0.66	330.20 ^c^ ± 2.18

Values in the same column marked with different letter differ significantly (*p* < 0.05).

**Table 6:** DPPH-free-radical-scavenging potential (% scavenging) and IC_50_ values for extracts from snacks without additive (3E), with 15% mulberry addition (9E), for mulberry (13E) and for GA (1 mM) (*n* = 4; ±SD).

Sample	DPPH	
	Scavenging [%]	IC_50_ [mg/mL] *
3E	50.54 ^a^ ± 2.52	36.62 ^a^ ± 3.09
9E	55.9 ^b^ ± 2.45	32.03 ^a^ ± 2.58
13E	87.45 ^c^ ± 3.58	3.33 ^b^ ± 0.26
Gallic acid	62.16 ^d^ ± 1.19	0.138 ^c^ ± 0.009

* IC_50_ values were calculated from dose–response curves and expressed as mg/mL of reaction mixture. Values in the same column marked with different letter differ significantly (*p* < 0.05).

**Table 7:** Inhibitory potential against AChE (after 15 min of reaction; extracts concentration 40 mg/mL) and for galantamine at concentration 1 mg/mL (*n* = 4, ±SD).

Sample	Inhibition [%] for Extracts
3E	59.88 ^a^ ± 1.81
9E	78.82 ^b^ ± 1.14
13E	79.37 ^b^ ± 0.65
Galantamine	85.52 ^c^ ± 4.52

Values in the same column marked with different letter differ significantly (*p* < 0.05).

**Table 8:** The total content of polyphenolic compounds (TPC mg GAE/g d.w.) for snacks with and without mulberry addition, and for mulberry before and after two-stage digestion (*n* = 4; ±SD).

Sample	Stage	TPC	Retention (%)
3E	Before digestion	0.20 ^a^ ± 0.011	100.0
	After I stage	0.11 ^b^ ± 0.005	55.0
	After II stage	0.04 ^c^ ± 0.002	20.0
9E	Before digestion	0.75 ^d^ ± 0.032	100.0
	After I stage	0.52 ^e^ ± 0.024	69.3
	After II stage	0.25 ^f^ ± 0.011	33.3
13E	Before digestion	4.0 ^g^ ± 0.231	100.0
	After I stage	2.8 ^h^ ± 0.124	70.0
	After II stage	1.3 ^i^ ± 0.051	32.5

Values in the same column marked with different letter differ significantly (*p* < 0.05).

**Table 9:** Concentrations (µg/g dry weight) of individual polyphenolic compounds in snacks without mulberry addition after two-stage digestion (*n* = 4; ±SD).

Compound	Before Digestion	Concentration After I Stage	Retention (%) After I Stage	Concentration After II Stage	Retention (%) After II Stage
Chlorogenic acid	0.34 ^a^ ± 0.031	0.12 ^b^ ± 0.005	35.3	0.05 ^c^ ± 0.002	14.7
Caffeic acid	0.13 ^a^ ± 0.012	0.04 ^b^ ± 0.002	30.8	0.02 ^c^ ± 0.001	15.4
Caffeoylglycerol	1.07 ^a^ ± 0.064	0.21 ^b^ ± 0.012	19.6	0.09 ^c^ ± 0.004	8.4
p-coumaroylquinic glycerol	2.28 ^a^ ± 0.101	0.45 ^b^ ± 0.025	19.7	0.33 ^c^ ± 0.022	14.5
Ferulic acid	1.30 ^a^ ± 0.083	0.46 ^b^ ± 0.024	35.4	0.25 ^c^ ± 0.012	19.2

Values in the same row marked with different letter differ significantly (*p* < 0.05).

**Table 10:** Concentrations (µg/g dry weight) of individual polyphenolic compounds in snacks with 15% mulberry addition before and after two-stage digestion (*n* = 4; ±SD).

Compound	Before Digestion	Concentration After I Stage	Retention (%) After I Stage	Concentration After II Stage	Retention (%) After II Stage
Dihydroxybenzoic acid hexoside	0.71 ± 0.02	-	-	-	-
Protocatechuic acid	24.00 ± 0.01	-	-	-	-
Neochlorogenic acid	3.22 ^a^ ± 0.06	0.91 ^b^ ± 0.032	28.3	0.35 ^c^ ± 0.011	10.9
Chlorogenic acid	7.29 ^a^ ± 0.22	2.25 ^b^ ± 0.082	30.9	0.94 ^c^ ± 0.035	12.9
Cryptochlorogenic acid	0.98 ^a^ ± 0.00	0.45 ^b^ ± 0.023	45.9	0.11 ^c^ ± 0.005	11.2
p-coumaroylquinic acid	0.29 ^a^ ± 0.01	0.15 ^b^ ± 0.006	51.7	0.06 ^c^ ± 0.003	20.7
Apigenin derivative	DNQ	-	-	-	-
Caffeic acid hexoside	DNQ	-	-	-	-
3,5-dicaffeoylquinic acid	0.29 ± 0.01	-	-	-	-
4,5-dicaffeoylquinic acid	DNQ	-	-	-	-
Rutoside	3.57 ^a^ ± 0.15	1.22 ^b^ ± 0.051	34.2	0.41 ^c^ ± 0.022	11.5
Quercetin dihexoside	0.54 ^a^ ± 0.03	0.15 ^b^ ± 0.006	27.8	-	-
Quercetin rhamnosyl dihexoside	1.08 ^a^ ± 0.01	0.25 ^b^ ± 0.013	23.1	0.09 ^c^ ± 0.004	8.3
Quercetin 3-O-glucoside	1.12 ^a^ ± 0.04	0.45 ^b^ ± 0.023	40.2	0.15 ^c^ ± 0.006	13.4
Quercetin acetylglucoside	1.7 ^a^ ± 0.03	0.71 ^b^ ± 0.034	41.8	0.28 ^c^ ± 0.012	16.5
Kaempferol 3-O-rutinoside	1.12 ^a^ ± 0.04	0.24 ^b^ ± 0.012	21.4	0.08 ^c^ ± 0.003	7.1
Kaempferol 3-O-glucoside	0.31 ± 0.00	-	-	-	-
Kaempferol derivative	DNQ	-	-	-	-

Values in the same row marked with different letter differ significantly (*p* < 0.05). DNQ—detected, not quantified.

**Table 11:** Concentrations (µg/g dry weight) of individual polyphenolic compounds in mulberry fruit before and after two-stage digestion (*n* = 3; mean ± SD).

Compound	Before Digestion	Concentration After I Stage	Retention (%) After I Stage	Concentration After II Stage	Retention (%) After II Stage
Dihydroxybenzoic acid hexoside	3.45 ^a^ ± 0.213	0.9 ^b^ ± 0.042	26.1	-	-
Protocatechuic acid	1.55 ± 0.11	-	-	-	-
Neochlorogenic acid	16.8 ^a^ ± 0.67	6.12 ^b^ ± 0.253	36.4	1.92 ^c^ ± 0.082	11.4
Chlorogenic acid	39.32 ^a^ ± 2.11	11.02 ^b^ ± 0.450	28.0	4.21 ^c^ ±0.174	10.7
Cryptochlorogenic acid	3.91 ^a^ ± 0.21	2.13 ^b^ ± 0.094	54.5	0.59 ^c^ ± 0.021	15.1
p-coumaroylquinic acid	1.09 ^a^ ± 0.08	0.57 ^b^ ± 0.024	52.3	0.21 ^c^ ± 0.013	19.3
Apigenin derivative	0.36 ^a^ ± 0.02	0.09 ^b^ ± 0.004	25.0	-	-
Caffeic acid hexoside	1.06 ^a^ ± 0.09	0.32 ^b^ ± 0.013	30.2	0.04 ^c^ ± 0.002	3.8
3,5-dicaffeoylquinic acid	1.89 ^a^ ± 0.11	0.50 ^b^ ± 0.023	26.5	0.11 ^c^ ± 0.005	5.8
4,5-dicaffeoylquinic acid	0.78 ^a^ ± 0.05	0.28 ^b^ ± 0.012	35.9	0.08 ^c^ ± 0.003	10.3
Rutoside	16.72 ^a^ ± 1.13	5.39 ^b^ ± 0.224	32.2	1.83 ^c^ ± 0.076	10.9
Quercetin dihexoside	2.26 ^a^ ± 0.13	0.76 ^b^ ± 0.034	33.6	-	-
Quercetin rhamnosyl dihexoside	5.19 ^a^ ± 0.31	1.57 ^b^ ± 0.066	30.3	0.52 ^c^ ± 0.021	10.0
Quercetin 3-O-glucoside	6.19 ^a^ ± 0.34	2.21 ^b^ ± 0.096	37.7	0.67 ^c^ ± 0.034	10.8
Quercetin acetylglucoside	8.62 ^a^ ± 0.41	3.64 ^b^ ± 0.142	42.2	0.98 ^c^ ± 0.042	11.4
Kaempferol 3-O-rutinoside	3.85 ^a^ ± 0.21	1.23 ^b^ ± 0.054	31.9	0.24 ^c^ ± 0.012	6.2
Kaempferol 3-O-glucoside	1.18 ^a^ ± 0.08	0.22 ^b^ ± 0.012	18.6	0.07 ^c^ ± 0.003	5.9
Kaempferol derivative	1.07 ^a^ ± 0.07	0.34 ^b^ ± 0.012	31.8	0.1 ^c^ ± 0.004	9.3

Values in the same row marked with different letter differ significantly (*p* < 0.05).

## Data Availability

The original contributions presented in the study are included in the article, and further inquiries can be directed to the corresponding author.

## Supplementary Materials

The following supporting information can be downloaded at: https://www.mdpi.com/article/10.3390/molecules31132370/s1, Table S1. Validation data used for quantitative analysis.
