# Supplementary material for: Development of Gluten-Free Corn Snacks Enriched with White Mulberry Fruit: Polyphenolic Composition, Antioxidant Activity and In Vitro Gastrointestinal Stability of Phenolic Compounds
Source: Molecules. 2026 Jul 5;31(13):2370. doi: 10.3390/molecules31132370 (PMC13363358; doi:10.3390/molecules31132370)
Supplement: Supplementary file 1 [file molecules-31-02370-s001.zip › molecules-4371461-supplementary.pdf]

Supplementary material

Development of Gluten-Free Corn Snacks Enriched with White Mulberry Fruit: Polyphenolic Composition, Antioxidant Activity and In Vitro Gastrointestinal Stability of Phenolic Compounds

Kamila Kasprzak-Drozd, Agnieszka Ziółkiewicz, Karolina Wojtunik-Kulesza, Marek Gancarz, Iwona Kowalska, Justyna Misiurek, Magdalena Wójciak, Ireneusz Sowa, Tomasz Oniszczuk, Maciej Combrzyński and Anna Oniszczuk

Table S1. Validation data used for quantitative analysis

Analyte	Wavelength	Concentration range	Equation	Correlation
Protocatechuic acid	260 nm	0.1-5.0 µg/mL	13.96x - 1.25	0.9994
Gentisic acid	325 nm	0.2-5.0 µg/mL	19.64x - 2.91	0.9988
Chlorogenic acid	325 nm	0.1-2.0 µg/mL	15.53x - 1.32	0.9985
Chlorogenic acid	325 nm	5.0-50 µg/mL	19.81x - 2.68	0.9986
p-coumaric acid	310 nm	0.2-5.0 µg/mL	54.53x - 0.13	0.9996
Ferulic acid	325 nm	0.2-2.0 µg/mL	60.44x - 0.42	0.9996
Caffeic acid	325 nm	0.1-2.0 µg/mL	44.61x + 7.53	0.9999
Kaempferol 3-*O*-rutinoside	350 nm	0.5-5.0 µg/mL	10.77x - 2.24	0.9991
Kaempferol 3-O-glucoside	350 nm	0.2-2.0 µg/mL	16.48x - 1.13	0.9987
Quercetin 3-*O*-rutinoside	350 nm	2.0-20 µg/mL	11.64x - 1.05	0.9989
Quercetin 3-*O*-rutinoside	350 nm	0.2-2.0 µg/mL	6.21x - 0.43	0.9992
Quercetin 3-O-glucoside	350 nm	0.5-10.0 µg/mL	20.36x - 0.89	0.9986
Apigenin 7-O-glucoside	350 nm	1.0-10 µg/mL	13.82x - 4.31	0.9996
3,5-dicaffeoylquinic acid	325 nm	0.2-2.0 µg/mL	12.38x + 7.91	0.9986
4,5- dicaffeoylquinic acid	325 nm	0.2-2.0 µg/mL	15.82x + 11.52	0.9972
